# Supplementary figures and images for: Genetic diversity and structure of the noble crayfish populations in the Balkan Peninsula revealed by mitochondrial and microsatellite DNA markers
Source: PeerJ. 2021 Aug 4;9:e11838. doi: 10.7717/peerj.11838 (PMC8349172; doi:10.7717/peerj.11838)

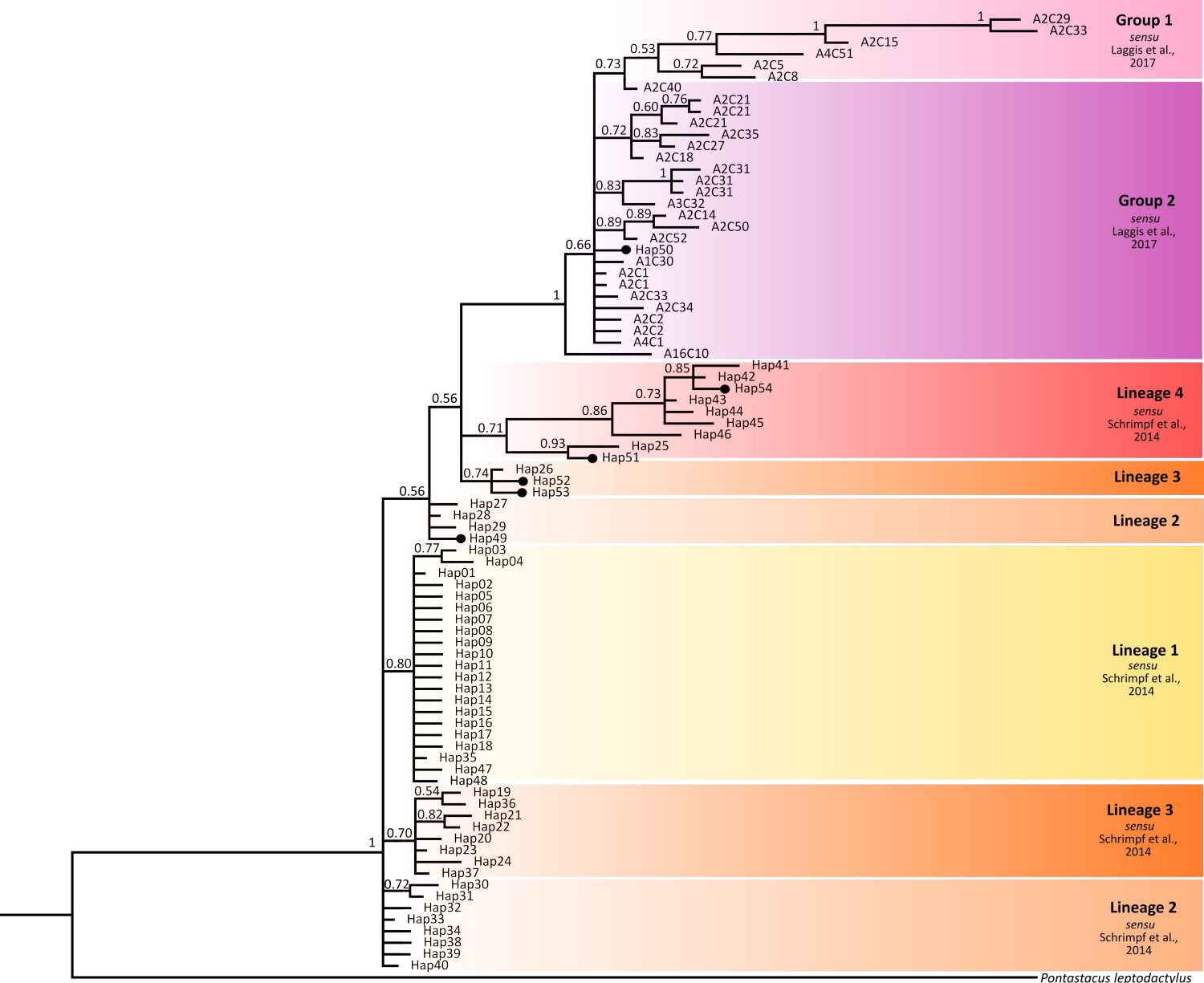

0.006

Supplement: Supplemental Information 2 — Values at nodes represent posterior probabilities >0.5. Phylogenetic clades are represented as in Schrimpf et al. (2014) (Lineages 1-4) and Laggis et al. (2017) (Group 1 and 2), and the position of new haplotypes is indicated by black dot at the end of branch. [file peerj-09-11838-s002.pdf]
